# Supplementary material for: Numerical modeling vs experiment of formic acid and formate ion behavior under gamma radiation at several pH values: Implications on prebiotic chemistry
Source: PLoS One. 2024 Dec 12;19(12):e0315409. doi: 10.1371/journal.pone.0315409 (PMC11637377; doi:10.1371/journal.pone.0315409)
Supplement: Attachment — Submitted filename: Response to Reviewers.docx [file pone.0315409.s001.docx]

Dear editor and anonymous reviewers:

We incorporate all the reviewers' comments and recommendations. We present a summary of all the changes and additions to the paper.

The changes for these comments are marked in red.

Reviewer #1

1) lines 140, 141, "Each coupled nonlinear differential equation system was solved using our Python (3.7.9) code...": Authors should report the full code as supporting information together with description on how to run the code, in order to allow the reproducibility of the results. Indeed, the latter is a mandatory requirement in any scientific work.

We have attached the link and cited the corresponding GitHub repository. Here, you can find the general algorithm to solve the system of differential equations based on a reaction mechanism and the full code of the formic acid model with the description and the instructions to run it.

Reviewer #2:

1) The title indicates only numerical modelling but not the experimental work. I'd suggest modifying it, perhaps something along the lines of "Numerical modeling vs experiment for formic acid..."

The text was changed as suggested.

2) on p 5, line 97, I'd change kinetics chemistry to chemical kinetics

The text was changed as suggested

3) On p. 6, some rationale is needed for the choices of these variables. They may make sense for a radiation-chemistry audience but the general PLOS ONE reader would need some indication of why those particular values are appropriate.

We add the corresponding justification in the beginning of the Methodology section.

4) On p. 7 just under Eq (1) something is wrong with " k_i,j^(i) is the rate constant for species j and k produced by the species i". There is no sub- or super-script k in Eq (10).

That is true, it was a mistake. We made corrections in the text.

5) Also, I think some general explanation of Eq (1) is needed.

We expand the explanation of Equation 1 below it.

6) On p. 7, I think a reference is needed for Eq 2, perhaps to a radiochemistry textbook?

We add the corresponding references.

7) On p. 15, the rationale for choosing k=10^4 seems weak to me. If the real values are greater than 10^4 then, yes, abrupt changes could occur, but they could be real. What would happen if k is less than 10^4?

We extend the rationale for choosing the rate constant *k=10^4^*, talking about numerical window stability in this part of the reaction chain, and deepen about the scenario if the *k* would be less or more than the stability interval.

8)on p. 18, what is the point of this "theoretical exercise". The rationale for substituting oxalic acid for CO2 is only given much later in the ms. Please bring this explanation up to the beginning of the Section so that the reader knows why you are doing this.

The text has been changed as suggested, and we extend the explanation about the objective of this theoretical model.

9) "at a pKa" isn't correct. pKa is a property of the acid, pH is the controlled quantity so pH should appear in the title and text, with an explanation early on that the chosen pH is equivalent to the pKa of formic acid.

Thank you so much for that observation. Title and text have been changed as suggested.

We are very grateful for all the comments and suggestions in this paper. They enrich each part and improve the quality of the work.

Yours sincerely, Alejandro Paredes-Arriaga.
